# Implementing a school-based HIV prevention program during public health emergencies: lessons learned in The Bahamas

**DOI:** 10.1186/s13012-022-01240-5

**Published:** 2022-10-04

**Authors:** Lynette Deveaux, Elizabeth Schieber, Lesley Cottrell, Regina Firpo-Triplett, Richard Adderley, Karen MacDonell, Nikkiah Forbes, Bo Wang

**Affiliations:** 1 Office of HIV/AIDS, Ministry of Health, Rosetta Street, Nassau, Bahamas; 2 Department of Population and Quantitative Health Sciences, UMass Chan Medical School, 368 Plantation Street, Worcester, MA 01605 USA; 3 Department of Pediatrics, West Virginia University, 959 Hartman Run Road, Morgantown, WV 26506 USA; 4 Dfusion Inc., 100 Enterprise Way, Suite D305, Scotts Valley, CA 95066 USA; 5 Department of Behavioral Sciences and Social Medicine, Florida State University College of Medicine, 1115 West Call Street, Tallahassee, FL 32306 USA

**Keywords:** Implementation fidelity, HIV prevention, Program resumption

## Abstract

**Background:**

Natural disasters and public health crises can disrupt communities’ capacities to implement important public health programs. A nationwide implementation of an evidence-based HIV prevention program, Focus on Youth in The Caribbean (FOYC) and Caribbean Informed Parents and Children Together (CImPACT), in The Bahamas was disrupted by Hurricane Dorian and the COVID-19 pandemic, especially in its more remote, Family Islands. We explored the teacher- and school-level factors that affected implementation of the program in these islands during those disruptions.

**Methods:**

Data were collected from 47 Grade 6 teachers and 984 students in 34 government elementary schools during the 2020–2021 school year. Teachers completed a pre-implementation questionnaire to record their characteristics and perceptions that might affect their implementation fidelity and an annual program training workshop. School coordinators and high-performing teachers acting as mentors received additional training to provide teachers with monitoring, feedback, and additional support. Teachers submitted data on their completion of the 9 sessions and 35 core activities of FOYC + CImPACT. The fidelity outcomes were the number of sessions and core activities taught by teachers.

**Results:**

On average, teachers taught 60% of sessions and 53% of core activities. Teachers with “very good” school coordinators (34% of teachers) taught more activities than those with “satisfactory” (43%) or no (34%) school coordinator (27.5 vs. 16.8 vs. 14.8, *F* = 12.86, *P* < 0.001). Teachers who had attended online training or both online and in-person training taught more sessions (6.1 vs. 6.2 vs. 3.6, *F* = 4.76, *P* < 0.01) and more core activities (21.1 vs. 20.8 vs. 12.6, *F* = 3.35, *P* < 0.05) than those who received no training. Teachers’ implementation was associated with improved student outcomes (preventive reproductive health skills, self-efficacy, and intention).

**Conclusions:**

The Hurricane Dorian and the COVID-19 pandemic greatly disrupted education in The Bahamas Family Islands and affected implementation of FOYC + CImPACT. However, we identified several strategies that supported teachers’ implementation following these events. Teacher training and implementation monitoring increased implementation fidelity despite external challenges, and students achieved the desired learning outcomes. These strategies can better support teachers’ implementation of school-based interventions during future crises.

Contributions to the literature

- Natural disasters and public health crises can interrupt implementation of evidence-based interventions (EBIs), and much of the literature on crises focuses on humanitarian efforts as opposed to resumption of EBIs.
- We evaluated strategies that supported teachers’ implementation of a school-based HIV prevention EBI in the Family Islands of The Bahamas after Hurricane Dorian and during the COVID-19 pandemic.
- Self-efficacy, teacher training, and school coordinators that provided monitoring and feedback increased teachers’ implementation fidelity and in turn student outcomes.
- These findings help fill gaps in the literature involving resumption of school-based EBI implementation during public health crises that disrupt their delivery.

## Background

Healthy People 2030 and similar strategic planning documents highlight select societal public health priorities (e.g., reducing HIV) that, if addressed, will significantly improve the health of citizens. Although these guidelines and planning efforts are key to coordinating resources, programming, and definitions of success, they are easily displaced in emergency situations. Natural disasters and other crises shift the attention of affected communities and can disrupt communities’ capacity to continue programing [1]. Much of the emergency response literature examines community response to the disaster itself, but it gives little attention to disruption of established programming.

Evidence-based interventions (EBIs) can be challenging to implement successfully in real-world settings [2]. With fewer than one-third of EBIs being adopted by the intended audience, it is important to consider program fidelity (i.e., the level to which facilitators adhere to the program, the dosage and quality of delivery, and participants’ responsiveness to program activities [3]) and attend to factors that contribute to successful adoption and implementation of a program. *Implementation Science* provides frameworks for evaluating implementation fidelity, adapting EBI and strategies to promote implementation, and engaging relevant collaborators, so researchers may analyze what factors affect these programs. Appropriate staff selection [4], sufficient training [5], continued facilitator supports such as coaching and mentorship [6], and monitoring and feedback [7] increase implementation fidelity and, in turn, lead to successful outcomes [8]. However, little is known about how natural disasters and other crises affect this process. We may assume that shifts in community capacity, resources, and priorities can negatively affect a program’s implementation. We need more information about the process that implementation teams experience when responding to crises or what efforts they might make to resume or sustain a program. Researchers have successfully adapted some EBIs in the face of the COVID-19 pandemic (e.g., type 2 diabetes mellitus intervention [9]; mental health therapy [10]), but there remains a need to examine the implementation strategies that support large-scale, school-based EBIs in times of crisis.

We explored and described experiences in implementing an adolescent sexual risk reduction program in the face of significant natural (Hurricane Dorian) and public health (COVID-19 pandemic) events. Implementation of an HIV prevention EBI in The Bahamas’ less populated Out Islands and Grand Bahama Island (for the sake of this paper, we will refer to these islands collectively as the “Family Islands”) during the 2019–2020 and 2020–2021 school years provided an opportunity to explore how these events changed the implementation landscape for the program.

*Focus on Youth in the Caribbean* (FOYC) plus *Caribbean Informed Parents and Children Together* (CImPACT) is an EBI designed to teach middle-school children how to avoid behaviors that spread HIV; it was implemented nationwide in The Bahamas using the Exploration, Preparation, Implementation, and Sustainment (EPIS) model [11] to enhance initial and sustained implementation. The EPIS framework describes factors that affect implementation throughout different stages of the implementation process. Our study focuses on the implementation and sustainment phases. Our partnerships with The Bahamas’ Ministry of Education (MOE) and Ministry of Health (MOH) served as the bridging factors between the “inner context” (e.g., individual teachers’ characteristics, school-level fidelity monitoring, peer mentoring) and the “outer context” factors (e.g., leadership, funding, school system-level environment, and networks). Our annual training and enhanced implementation strategies (biweekly monitoring and feedback (BMF) and site-based assistance and mentorship (SAM)) addressed factors of these inner and outer contexts to support teachers’ implementation fidelity.

The Family Islands of The Bahamas experienced worse effects of Hurricane Dorian and had different access to resources during COVID-19 school closures than the most populated island, New Providence. These differences allowed a unique opportunity to examine the factors that affected program implementation during times of crisis and whether our evidence-based, culturally validated implementation strategies were robust enough to support implementation fidelity in the more disadvantaged Family Islands. Specifically, our goal was to examine adherence to the original program implementation plan, while teachers and the larger community experienced these significant public health events.

### Natural disasters’ disruption of education and downstream health effects

Tropical cyclones are increasing in severity [12], and they disrupt communities and schools in many ways. Following Hurricane Katrina, teachers in southern Louisiana reported that displaced students, lack of school resources and supports, and negative psychological effects on students and teachers were major barriers to education [13]. Despite historical examples of schools’ importance and needs following major hurricanes, areas have continued to be ill-prepared for these extreme weather events (e.g., Hurricanes Sandy, Maria, Dorian) [14], which can have negative effects on students’ performance in school [15]. In addition to fiscal costs, school interruptions, and acute dangers, hurricanes are linked to long-term health effects. HIV, syphilis, and gonorrhea positivity rates increased in New Orleans’ sexual health clinics following Hurricane Katrina and the increase in gonorrhea rates in observed high schools more than doubled [16]. Schools provide opportunities to help students recover from acute disasters, but they need to be equipped to adapt to the immediate needs of the students and educators.

In 2019, Hurricane Dorian caused widespread damage in the Family Islands of The Bahamas, specifically Grand Bahama and Abaco. The costs of damage to the education sector alone were US $72.4 million [17]. Seven schools were destroyed, and an additional 38 were damaged, displacing 1500 students and 120 teachers. The damage and lack of potable water led to school closures and loss of instructional time during the first semester of school, when FOYC is typically implemented in the Grade 6 classrooms. This loss delayed implementation of FOYC+CImPACT. Additionally, when schools reopened in October 2019, implementation in these islands was further delayed because of administrations’ and teachers’ curricula priorities, availability of resources to complete program activities, and the time needed to return to a semblance of normalcy. Unfortunately, The Bahamas was not afforded much time to recover before the next crisis affected its schools.

### Effects of COVID-19-related school closures and travel mandates

Six months after Hurricane Dorian decimated parts of The Bahamas, the Ministry of Education (MOE) closed all public and private schools on March 16, 2020, because of the COVID-19 pandemic [18]. Initially, the closure was for one month with the intention to return to in-person classes, but instruction resumed via virtual formats for the remainder of the 2019–2020 school year and fluctuated between face-to-face, virtual, and hybrid formats through the 2020–2021 school year. Decisions to have face-to-face or virtual instruction were dictated per island by the Ministry of Health (MOH) and were based on current COVID-19 cases and schools’ capacities to use virtual learning platforms. Virtual instruction, though safer during a pandemic, introduced significant challenges for Bahamian teachers and students. In addition to technology accessibility, teachers had to translate all instructions to a virtual format, including the FOYC+CImPACT program. Another large barrier for implementation in the Family Islands was the travel ban between islands. A series of nationwide lockdowns and curfews commenced on March 23, 2020, and resulted in no interisland travel. When travel to the islands resumed, a 14-day quarantine was imposed on all travelers. Thus, the FOYC team was unable to travel to the more remote Family Island schools, conduct community forums (for community buy-in), train teachers in-person to implement the program, or use the enhanced implementation strategies as they had in years past [19].

The COVID-19 pandemic changed the way the world operates and has had huge impacts on the education system. Many countries suspended face-to-face instruction and moved instruction to virtual formats [20]. Curricula were prioritized differently with time reallocated to remote instruction [20], but that does not reduce the importance of topics addressed by school-based EBIs. Additionally, teachers with higher technology competence and technology-specific training opportunities were more successful at delivering their curricula in an online format during COVID-19 closures [21], but schools may not have been able to provide adequate training because of the rapid onset of the pandemic. The entire education system needs to maintain flexibility with continued virtual instruction, hybrid models, and resumption of face-to-face instruction, and EBIs need to fit into the instructional modes. Staff training is essential for implementation of school-based programs [4], and it can and should be used to prepare staff to implement EBIs in the current educational landscape.

### Research questions

Toward the goal of investigating what factors increased or impeded implementation of FOYC+CImPACT in the Family Islands of The Bahamas, we asked the following research questions:

1. To what extent were the enhanced implementation strategies (i.e., BMF and SAM) used in the 2020–2021 school year amidst school closures?
2. To what extent did teachers implement the curricula with fidelity?
3. What teacher- and school-level factors affected implementation fidelity following Hurricane Dorian and the onset of the COVID-19 pandemic?

## Method

### Study site

The original research plan was to implement FOYC+CImPACT with enhanced implementation strategies nationwide in the 2019–2020 school year. Because of the damage caused by Hurricane Dorian and the school closures related to the COVID-19 pandemic, it was decided that implementation in the Family Islands would begin in the 2020–2021 school year, resulting in two waves of implementation: wave 1 in New Providence and wave 2 in the Family Islands. Thirty-four elementary schools in the Family Islands of The Bahamas participated in the second wave of the national implementation of FOYC+CImPACT in the fall of 2020. A total of 47 Grade 6 teachers and 984 students participated in the study. The research protocol was approved by the UMass Chan Medical School Institutional Review Board and the Institutional Review Board of the Bahamian Princess Margaret Hospital, Public Hospitals Authority.

### FOYC+CImPACT program

Teachers implemented the 8-session FOYC adolescent HIV prevention curriculum in Grade 6 classrooms. The curriculum includes 30 core activities. FOYC is incorporated in the Health and Family Life Education (HFLE) curriculum for The Bahamas. The CImPACT parental monitoring intervention is a single session (with 5 core activities) between parents, students, and teachers. School coordinators provided teachers BMF, and high-performing teachers acted as peer mentors to provide SAM; these enhanced implementation strategies were chosen based on empirical evidence that implementation monitoring and feedback as well as mentorship and coaching are effective strategies to promote implementation fidelity [7]. They were developed with the local research team and teachers to be culturally sensitive and to address inner and outer contextual factors of the EPIS framework. Further, school coordinators communicated with the FOYC research team to identify issues that could be addressed by leaders and policies (e.g., online data collection) in the outer context. These enhanced implementation strategies were shown to be effective for supporting FOYC implementation fidelity prior to waves 1 and 2 of the national implementation and have been described in detail elsewhere [19]. Initially, each school was supposed to be assigned a school coordinator and have mentors. However, because of barriers imposed by the crises, only 32% of schools had coordinators, and only 24% had mentoring teachers for the 2020–2021 school year.

### Teacher training

Typically, the annual FOYC+CImPACT teacher training workshops were two-day, in-person sessions led by three Bahamian FOYC trainers and a US training specialist with extensive experience with FOYC+CImPACT. The training followed FOYC guidelines and consisted of clear objectives, short lectures, interactive group discussions, videos, demonstrations of curricula activities and skills, skill practice, role play, and teach backs [22]. The workshop aimed to increase participants’ curriculum knowledge, strengthen teacher attitudes about the positive effects of the curriculum, and improve teachers’ skills and comfort with the curriculum. Specifically, teachers (1) reviewed the need for HIV prevention in The Bahamas, (2) received an overview of FOYC+CImPACT and its past efficacy, (3) observed models of the 30 core activities in the eight sessions of FOYC, (4) participated in a didactic question-and-answer period about puberty and contraception and condom use, (5) observed a model of CImPACT, and (6) received in-depth skill instruction and practice teaching one or more core activities with live feedback. Teachers also received examples of approaches to address the curriculum in their classrooms, as well as copies of the FOYC teacher training manual and FOYC+CImPACT 24/7 flash drives for remote, digital “point-of-care” implementation guidance.

In the fall of 2019, 33 teachers completed the training workshops in Grand Bahama prior to the arrival of Hurricane Dorian. After the hurricane and then the onset of the COVID-19 pandemic, all trainings and plans for implementation of the curriculum in the Family Islands were suspended until the 2020–2021 school year.

Teacher training webinars for 2020–2021 school year all took place synchronously on a virtual platform. Twenty-eight Family Island teachers registered for the webinars in advance and completed the pre-workshop measures and consent. The webinars consisted of three 2-h sessions, which consisted of live, instructor-led trainings designed to provide new FOYC teachers with relevant curriculum information and resources required to implement FOYC+CImPACT. Trainers modeled the core activities and sessions of the curriculum. Teachers also received a summary of the FOYC efficacy research and a situational analysis of HIV/AIDS and teen pregnancy in The Bahamas. Trainers conducted didactic and interactive sessions to increase teachers’ comfort levels with the material and answered sensitive questions that could arise in the classroom. Teachers received an electronic copy of the manual and resources. Teachers could access recordings of the webinars to review the sessions, and teachers who were unable to attend had access to the recordings.

### School coordinator and mentor training

A school coordinator was identified for 11 of the 34 schools to complete BMF. Most of the coordinators were guidance counselors or vice principals who oversaw all Grade 6 HFLE teachers within a school and could coordinate between classrooms and monitor activities. Teachers’ implementation and progress were monitored biweekly over the course of the school year via a standard form where coordinators noted the date(s) of the sessions, how many core activities were completed, recorded notes for the teachers to review as feedback, and noted any issues teachers have had (such as scheduling or difficulty translating particular core activaties to virtual formats) to report to the FOYC research office. Eight high-performing teachers and guidance counselors provided SAM to at-risk and moderate-performing teachers who had been identified by their responses on our validated Pre-implementation Screening Tool [23] and their implementation performance in the past year. Mentors were trained to identify challenges, assist teachers in preparing sessions, model how to teach the core activities, and provide guidance for curriculum delivery. Four Bahamian trainers with extensive experience with FOYC+CImPACT conducted 2- to 3-h training sessions with school coordinators and mentors in 2019.

### Measures

### Implementation fidelity

Implementation fidelity was defined as number of the 35 core activities of the curriculum taught. The degree of implementation was the number of the nine sessions covered. Teachers completed a Teacher Implementation questionnaire specific to the FOYC+CImPACT sessions following each session. They documented the activities covered in each session, their degree of comfort with the session (very comfortable, somewhat comfortable, and not comfortable at all), whether they taught the activities as outlined in the manual or made any modifications to the format of activities in the manual, and how many students (most, some, few) seemed engaged in the session.

#### Teachers’ characteristics, training experience, and perceptions

Most teachers (*n* = 45) completed a pre-implementation questionnaire to provide information known to affect implementation fidelity [23]: level of formal education; years as a teacher; attendance at the training workshop, either in years past or for the current school year; perceptions of the importance of HIV prevention (“very meaningful” to “not at all meaningful”) for Grade 6 students; comfort level teaching the FOYC+CImPACT curriculum; and any competing lessons or teaching priorities. The pre-implementation questionnaire consisted of four items assessing teachers’ perceived *principal supportiveness* (e.g., “My principal is supportive of teaching FOYC”) [24, 25]; eight items assessing teachers’ *attitudes toward sex education in schools* (e.g., “Young people are given too much information on sex”) [26]; three items assessing their *self-efficacy* in teaching the FOYC+CImPACT curriculum (e.g., “I feel like I can teach the FOYC+CImPACT program according to the manual”) [27]; four items assessing teachers’ *autonomy* in their classroom (e.g., “I determine norms and rules for student behavior within my classroom”) [28]; and five items assessing teachers’ *confidence* teaching five topics: condom use, teen pregnancy, alcohol and drug use, sexual harassment/abuse, and HIV/AIDS [29]. Each item used a 5-point Likert scale (1 = “totally disagree” to 5 = “totally agree” for principal supportiveness, attitudes toward sex education, self-efficacy, and autonomy and 1 = “not at all confident” to 5 = “very confident” for confidence). The internal consistency (Cronbach’s α) of the scales is adequate (principal supportiveness, *α* = 0.77; attitudes toward sex education, *α* = 0.72; self-efficacy, *α* = 0.73; autonomy, *α* = 0.77; confidence, *α* = 0.85).

The national coordinator and The Bahamas’ FOYC project manager, as well as FOYC curriculum trainers, assessed the performances of school coordinators and mentors with a brief survey. The survey evaluated 14 items as “unsatisfactory,” “satisfactory,” “good,” or “excellent,” including coordinator’s knowledge of the FOYC curriculum, communication with the research office, perception of leadership, submission of measures, number of sessions mentored, and whether the mentor was remote or site based. Assessors also noted school coordinators’ and mentors’ performance via free responses.

#### Student outcomes

Students completed an anonymous curricular assessment instrument, adapted by the MOE from a version of the Bahamian Youth Health Risk Behavioral Inventory [30], prior to experiencing FOYC in grade 6, and they repeated the assessment at the end of Grade 6 (6 months after exposure to the curriculum). Their data were deidentified and aggregated by classroom and then linked to teachers’ performances. The instrument assessed HIV/AIDS knowledge, preventative reproductive health skills, and students’ perceptions of self-efficacy, intentions, and self-reported behaviors. HIV/AIDS knowledge was assessed via 16 “true or false” statements. Preventative reproductive health skills were assessed with an adaptation of the Condom-use Skills Checklist [31], which consisted of six true or false statements that describe correct condom use. Students’ self-efficacy to practice safe sex was assessed with five items on a 5-point Likert scale (1 = “strongly disagree” to 5 = “strongly agree”), and the mean was used as a composite score. The internal consistency of the scale was 0.78. Intention to use condoms for protection was assessed with a question “what are the chances that you would use a condom if you need to prevent yourself from getting HIV” and which students ranked on a 5-point Likert scale (1 = “no chance in the world” to 5 = “yes, big chance that I would”).

### Analysis

First, frequency distributions summarized the number of sessions taught (of the nine possible) and the number of core activities completed (of the 35 possible). Second, to identify factors associated with teachers’ fidelity of implementation, we used ANOVA (and Student’s *t*-test) to relate the number of core activities and the number of sessions taught to teachers’ personal characteristics, training experience, and perceptions. We also examined association of initial teacher training (in-person, online, and both) and continued implementation support (implementation monitoring and peer mentoring) with implementation fidelity. Third, we examined the difference between baseline and follow-up in students’ HIV/AIDS knowledge, preventive reproductive health skills, self-efficacy, and intention to use protection (using Student’s *t*-test). The test statistics were adjusted for the clustering effects of classroom and/or school using variance inflation factors (VIFs). The analyses used SAS 9.4 statistical software (SAS Institute Inc., Cary, NC, USA).

## Results

### Teachers’ implementation fidelity

**Fig. 1:**
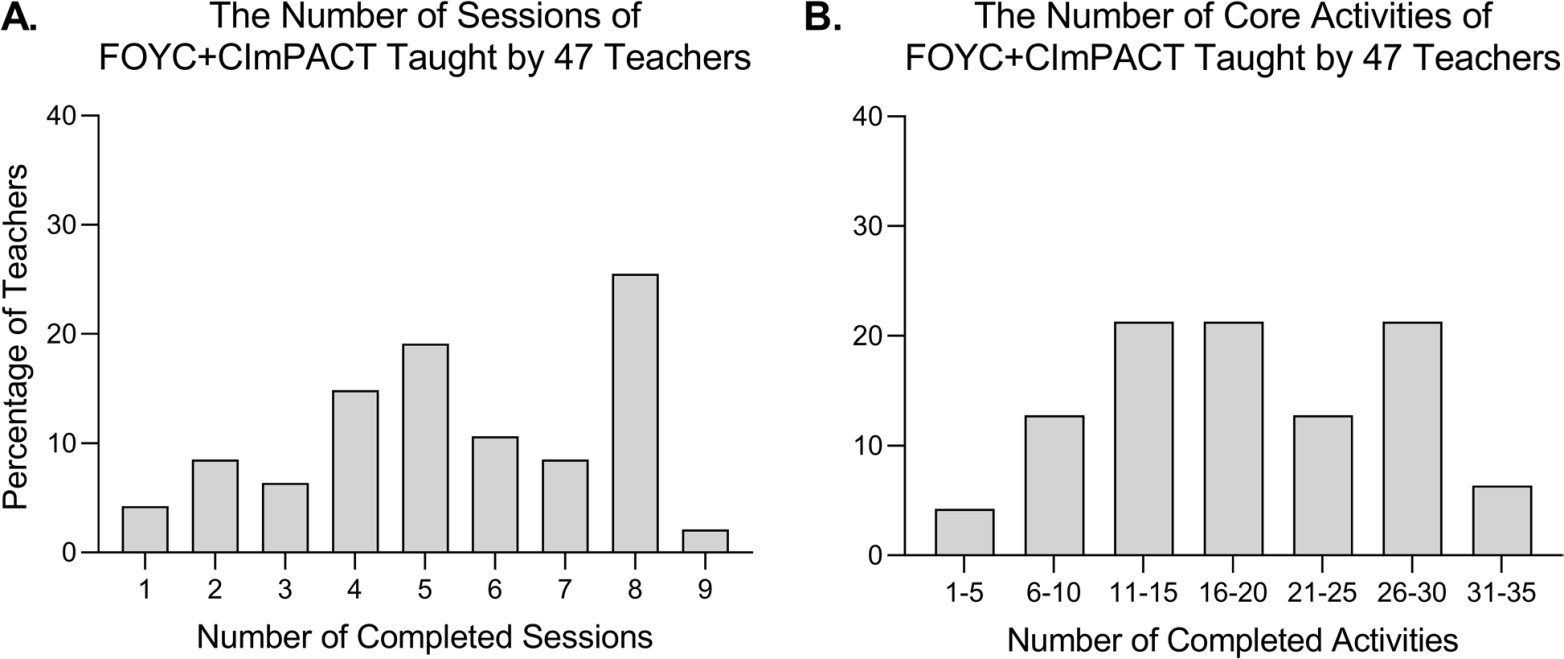
Percentage of teachers who taught each number of sessions (**A**) and core activities (**B**)

Figure 1 displays a histogram of the numbers of sessions (A) and core activities (B) completed by teachers. On average, teachers taught 5.4 of 9 sessions (60%) and 18.6 of 35 core activities (53%). Of the 47 teachers, 13 (28%) completed 25 or more core activities and covered eight or nine sessions, but six (13%) taught only one or two sessions. Thirty-one (66%) taught 5 or more sessions, and twenty-nine (62%) taught 16 or more core activities—equivalent to half of the intervention curriculum. One teacher taught the CImPACT session and 4 of 5 core activities, the parent component of the intervention.

### Association of teachers’ characteristics, training experience, perceptions of HIV prevention, and FOYC implementation fidelity

Table 1 presents the average number of FOYC core activities and sessions taught by teachers with various personal characteristics and perceptions. The CImPACT session and activities were not included in this analysis because only one teacher delivered the session. Teachers who perceived that their principal believed teaching FOYC was important taught more core activities exactly as outlined in the manual than teachers who did not know how their principal felt (13.6 vs. 8.0, *t* = 2.27, *P* < 0.05). Teachers with prior experience teaching an HIV prevention program were marginally associated with number of core activities taught as outlined in the manual. Other teacher characteristics and perceptions were not associated with implementation.

**Table 1:** Association between teachers’ characteristics, teaching experience, perceptions of HIV prevention, and number of FOYC + CImPACT core activities and sessions completed

Variables	*n*	Number of core activities completed (0–35)	Number of core activities taught exactly as outlined (0–35)	Number of sessions taught (0–9)
Total sample Education level	45			
Certificate/associate degree	6	23.0 (6.8)	7.3 (6.5)	6.8 (1.5)
Bachelor degree	30	18.0 (8.0)	12.8 (7.6)	5.4 (2.1)
Master degree	9	20.4 (8.5)	13.8 (7.1)	5.6 (2.2)
Perceptions of importance of HIV prevention programs for grade 6				
Very important	38	19.1 (7.8)	12.3 (7.6)	5.6 (2.1)
Somewhat important	7	19.4 (9.8)	12.1 (7.1)	5.7 (2.1)
Prior experience of teaching HIV risk reduction intervention				
Yes	6	22.5 (5.4)	17.2 (4.4)#	6.3 (1.5)
No	39	18.6 (8.2)	11.5 (7.6)#	5.5 (2.2)
Having other teaching priorities				
Yes	12	19.3 (8.2)	12.2 (8.7)	5.7 (2.3)
No	33	19.1 (8.0)	12.3 (7.1)	5.6 (2.0)
FOYC is a Bahamian curriculum				
Very much so	23	20.4 (7.9)	12.7 (7.4)	6.0 (2.0)
Somewhat	17	16.9 (8.5)	11.4 (7.0)	5.0 (2.2)
What do you think your school principal thinks about teaching FOYC in grade 6?				
I think my principal believes it is important to teach FOYC	34	19.9 (8.4)	13.6 (7.3)*	5.8 (2.2)
I do not know what my principal thinks about my teaching FOYC	11	16.6 (6.3)	8.0 (6.5)*	4.9 (1.5)
Training in interactive teaching				
A lot	10	17.3 (8.4)	13.9 (7.6)	5.0 (2.3)
Some	15	20.2 (6.3)	13.4 (6.8)	6.1 (1.6)
A little or none	20	19.3 (9.0)	10.6 (7.8)	5.5 (2.3)

#*P* < 0.10; *P < 0.05. Scores reported as mean (standard deviation)

### Association of initial teacher training and implementation supports with implementation fidelity for FOYC+CImPACT

As shown in Table 2, teacher training modality and the performance of school coordinators were significantly associated with teachers’ implementation fidelity. Teachers who attended the online training webinar taught, on average, the most core activities (21.1) followed by those who attended in-person and online trainings (20.8), and in-person training only (19.4), whereas teachers who received no training taught the fewest core activities (12.6; *F* = 3.35, *P* < 0.05). Similarly, teachers who attended online and in-person trainings, online only, and in-person only taught more sessions on average than teachers who received no training (6.2 vs 6.1 vs 5.7 vs 3.6; *F* = 4.76; *P* < 0.01). Teachers who had “very good” school coordinators on average taught the most sessions, followed by teachers with “satisfactory” school coordinators and then teachers without coordinators (7.7 vs. 5.1 vs. 4.3; *F* = 12.88; *P* <0.001). Teachers with “very good” school coordinators also covered the most core activities, followed by teachers with “satisfactory” coordinators then teachers without school coordinators (27.5 vs. 16.8 vs. 14.8; *F* = 12.86; *P* < 0.001).

**Table 2:** Association between teacher training and continued implementation support with teachers’ implementation fidelity

Variables	*n*	Number of core activities completed (0–35)	Number of core activities taught exactly as outlined (0–35)	Number of sessions taught (0–9)
Total sample	47	18.6 (8.3)	11.8 (7.7)	5.4 (2.2)
Teacher training				
In-person	7	19.4 (8.4)	9.3 (7.8)	5.7 (2.4)
Online	17	21.1 (8.7)*	12.8 (8.4)	6.1 (2.1)**
Both in-person and online	11	20.8 (6.8)	15.5 (5.8)	6.2 (1.6)**
No training	12	12.6 (6.4)*	8.3 (6.7)	3.6 (1.9)**
Performance of school coordinators				
No school coordinator	16	14.8 (6.9)***	10.5 (6.7)	4.3 (1.8)***
Satisfactory	20	16.8 (7.5)***	11.0 (7.5)	5.1 (2.1)***
Very good	11	27.5 (4.3)***	15.0 (9.0)	7.7 (0.9)***
Performance of peer mentors				
No mentor	40	18.4 (8.6)	11.7 (7.6)	5.3 (2.3)
Satisfactory	7	19.9 (6.6)	12.3 (8.6)	6.0 (1.7)

*P < 0.05; **P < 0.01; ***P < 0.001. Scores reported as mean (standard deviation)

### Correlation among factors influencing teachers’ implementation degree and fidelity

Pearson correlation coefficients were used to determine the strength of associations between factors influencing teachers’ self-efficacy and implementation (Table 3). Teachers’ comfort level with the curriculum, confidence in implementing core activities, attitudes toward sex education in schools, autonomy, and perceived principal support were significantly related to increased self-efficacy (*r* = 0.32–0.70; *P* < 0.05), which was in turn significantly related to the number of core activities taught as outlined in the manual (*r* = 0.61; *P* < 0.001). Teachers’ comfort level with the curriculum was also significantly related to their confidence in implementing core activities, attitudes toward sex education, perceived principal support, and the number of core activities taught as outlined in the manual (*r* = 0.41–0.60; *P* < 0.01). Teachers’ confidence in implementing core activities was also significantly related to their attitudes toward sex education in schools and the number of core activities taught as outlined in the manual (*r* = 0.44 and 0.35; *P* < 0.05). Teacher’s attitudes toward sex education in schools were significantly related to their perceived principal supportiveness and the number of core activities taught as outlined (*r* = 0.29 and 0.33; *P* < 0.05). Teachers’ autonomy was related to perceived principal support (*r* = 0.34; *P* < 0.05), and perceived principal support was related to the number of activities taught (*r* = 0.29; *P* < 0.05). The number of core activities taught and the number of activities taught exactly as outlined in the manual were also related (*r* = 0.61; *P* < 0.001), and the number of sessions completed was significantly related to the number of core activities taught (*r* = 0.95; *P* < 0.001) as well as those taught as outlined in the manual (*r* = 0.55; *P* < 0.001).

**Table 3:** Bivariate correlation among factors influencing teachers’ self-efficacy and implementation

Variables	1	2	3	4	5	6	7	8	9	Mean	SD
1. Comfort level with the curriculum	1.00									2.30	0.57
2. Confidence in implementing core activities	0.60c	1.00								4.29	0.65
3. Attitudes toward sex education in schools	0.59c	0.44b	1.00							3.69	0.63
4. Autonomy	0.14	−0.13	0.20	1.00						4.01	0.63
5. Perceived principal support	0.41b	0.23	0.29a	0.34a	1.00					3.77	0.67
6. Self-efficacy	0.70c	0.34a	0.47b	0.32a	0.45b	1.00				3.41	0.86
7. Number of core activities taught	0.24	0.09	0.20	−0.03	0.29a	0.28	1.00			18.60	8.27
8. Number of core activities taught exactly as outlined in the manual	0.50c	0.35a	0.33a	−0.08	0.26	0.61c	0.61c	1.00		11.77	7.65
9. Number of sessions completed	0.21	0.14	0.23	−0.05	0.27	0.18	0.95c	0.55c	1.00	5.43	2.20

^a^*P* < 0.05

^b^*P* < 0.01

^c^*P* < 0.001. *SD* standard deviation. Score range: 1~5 for confidence, sex education, principal support, and self-efficacy

### Student outcomes

Table 4 summarizes the baseline and follow-up scores on students’ curricular assessments. After adjusting test statistics with VIFs, students showed significant gains in all four areas. Their HIV/AIDS knowledge increased from a score of 8.0 (out of 15) to 10.3 (*t* = 7.98; *P* < 0.001). Preventative reproductive health skills increased from a score of 3.6 (out of 6) to 4.0 (*t* = 2.90; *P* = 0.004). Their self-efficacy increased from a score of 2.3 (out of 3) to 2.7 (*t* = 5.48; *P* < 0.001). Finally, their intention to use protection increased from 2.6 (out of 5) to 3.4 (*t* = 4.99; *P* < 0.001).

**Table 4:** Students’ HIV/AIDS knowledge, preventive reproductive health skills, self-efficacy, intention to use condoms at baseline, and 6-month follow-up

	Baseline	Follow-up	*Adj*. t	*p*
Sample size	885	984		
HIV/AIDS knowledge (scores 0–15)	8.00 (3.62)	10.25 (3.04)	7.98	< 0.001
Preventative reproductive health skills (scores 0–6)	3.60 (1.61)	3.98 (1.57)	2.90	0.004
Self-efficacy (scores 0–3)	2.27 (1.10)	2.72 (0.99)	5.48	< 0.001
Intention to use protection (scores 1–5)	2.62 (1.97)	3.41 (1.89)	4.99	< 0.001

Test statistics (*t*-values) were adjusted using variance inflation factors (VIFs). Scores reported as mean (standard deviation)

## Discussion

FOYC+CImPACT is poised to make significant contributions to school-based HIV prevention and implementation science, through the commitment and ongoing involvement of both the Ministry of Education (MOE) and Ministry of Health (MOH) in The Bahamas and its adoption in China, Vietnam, and Namibia. Having been developed, tailored to The Bahamas, and implemented for over 20 years, it has achieved high levels of successful implementation [16] and student outcomes [19, 32, 33]. However, implementation fidelity in the Family Islands was affected by Hurricane Dorian and the COVID-19 pandemic. External challenges such as school closures and travel restrictions served as barriers to teachers’ implementation fidelity. For example, only one teacher in our study was able to host the CImPACT session during the 2020–2021 school year because travel restrictions and social-distancing considerations prevented teachers from hosting in-person parent-teacher meetings, and adapting the CImPACT session to a virtual format was not a priority that school year. However, our analyses show that the implementation strategies, when available, were successful at promoting teachers’ implementation fidelity of FOYC despite these externalities.

School coordinators’ performance, teacher training workshops and webinars, and administrative support (i.e., principals) were all positively associated with teachers’ implementation fidelity. With inner context factors such as self-efficacy, comfort with the curriculum, confidence in implementing core activities, and teacher attitudes being significantly related to teaching the core activities as outlined in the manual, it is apparent that increasing these factors was important for supporting teachers’ quality of delivery, which is consistent with prior research of the EPIS model [34]. School coordinators’ performance was significantly associated with the number of core activities and sessions completed but not associated with the number of core activities taught exactly as outlined. This finding may suggest that monitoring alone increased teachers’ implementation, but it was teachers’ skills that enhanced the quality of their delivery. Our training workshops, school coordinator activities, and peer mentors’ efforts were designed to promote teachers’ skills and provide monitoring, addressing the combined factors that were related to implementation fidelity and quality. The average implementation fidelity in the Family Islands for the 2020–2021 school year was lower than implementation fidelity in New Providence (*M* = 25.1 core activities taught, *SD* = 5.1 activities). New Providence was less affected by the Hurricane Dorian and had better access to supports during the COVID-19 pandemic. Further, the FOYC+CImPACT with BMF and SAM effectiveness trial was conducted in New Providence, and wave 1 rollout occurred during the 2019–2020 school year; thus, New Providence teachers received training and enhanced implementation supports before the Family Island teachers. However, implementation fidelity in the Family Islands was greater with our enhanced strategies than previous, standard implementations (teacher training only). From 2011 to 2016, teachers in the Family Islands taught 16.3 core activities (*SD* = 8.7) and 4.4 sessions (*SD* = 2.3) [35], while teachers in the 2020–2021 school year taught 18.6 core activaties and 5.4 sessions.

Although the dual crises in The Bahamas were unprecedented, and interruptions in the school system were extensive, teachers were able to resume implementing FOYC. These types of crises have different effects on school-based EBI implementation. The Hurricane Dorian caused acute damage and immediate displacement of teachers and students, which stressed the affected schools and delayed reopening of schools, while the COVID-19 pandemic caused the education sector system-wide disruptions and long-lasting effects. However, despite these combined crises, we found our implementation strategies, when available, promoted teachers’ implementation fidelity upon the program’s resumption. Thus, this experience highlights the importance of planning for implementation strategies that are adaptable to changing circumstances.

### Need for disaster preparedness

The combination of Hurricane Dorian and the COVID-19 pandemic in The Bahamas Family Islands demonstrated the importance of being prepared to mitigate and respond to disasters. Extreme weather events are inevitable and are increasing in severity [12]. Of the five category, 5 hurricanes that made landfall in the Atlantic basin since 2016, two struck The Bahamas [36]. Climate change will continue to worsen the intensity of tropical cyclones [37] and as these storms will have far-reaching effects on coastal areas and especially small island nations. Beyond structural damage and financial strain, these storms can lower people’s access to healthcare and education, drive migration, and affect people’s mental health [36]. Similarly, experts have monitored and attempted to prepare for the next pandemic for years [38], but the world was still widely unprepared for the COVID-19 pandemic. While virologists work to monitor zoonotic risks of spillover events to predict potential epidemics and pandemics [39], implementation scientists should use the lessons learned during the COVID-19 pandemic to create more robust systems for EBIs.

### Adaptability and future directions

During the pandemic, synchronous online trainings replaced in-person trainings effectively. Teachers who attended only online training for FOYC+CImPACT had similar implementation fidelity to those who have previously attended in-person training workshops, and they taught significantly more sessions and core activities than those who received no training. It is important to note that teachers that attended an in-person training workshop but not the online training had a delay of at least 1 year between attending that training and implementing FOYC+CImPACT because implementation was suspended in the 2019–2020 school year. This delay between training and implementation could have reduced the efficacy of the in-person training. Thus, we cannot directly compare the efficacy of the online webinars versus the in-person training with these analyses. However, we can say the online training was related to teachers’ implementation fidelity in the 2020–2021 school year. Other researchers have had similar findings regarding online training. Researchers trained teachers to implement a life skills EBI through in-person or virtual modalities and found that those who received only online training rated the quality of the training lower, but the implementation quality in their classrooms did not differ from those that attended in-person trainings [40]. Thus, online training has the potential to be just as effective in preparing teachers to implement an EBI, and it affords more flexibility for teachers and trainers to attend safely, at their convenience (if the material is available asynchronously), and/or save travel costs, all of which provide a level of flexibility to mitigate barriers posed by crises. Future studies should further explore the relative efficacy of different modalities of teacher training for school-based EBI implementation.

If researchers and program facilitators are better prepared to respond to challenges posed by crises, implementation of school-based programs will be more sustainable and effective, even in the face of adversity. In the education sector, it is of the upmost importance to provide strong training for teachers and program facilitators. Darling-Hammond and Hyler stressed the importance of preparation and the ability to pivot training to educators’ current needs (e.g., how to teach virtually) to increase teachers’ self-efficacy and performance [41]. They also recommended creation and/or use of peer mentoring and collaboration to provide educators more supports to increase their capacity to adapt to the strains posed by crises. These recommendations are similar to our enhanced implementation strategies for FOYC+CImPACT. The FOYC team was not prepared for Hurricane Dorian’s effects and for a global pandemic to change the education system within a school year. However, our strategies could theoretically mitigate some of the effects of future crises on EBI implementation fidelity. The responses to Hurricane Dorian and the COVID-19 pandemic in The Bahamas can serve as learning opportunities and allow us to be better prepared to address future challenges.

### Limitations

Beyond the challenges posed by travel restrictions and school closures, there were several limitations to this study. The data collected were self-reported by the teachers and thus are subject to reactivity and response bias. Typically, trained observers attend 10% of teachers’ sessions, and agreement between teachers and observers has been high (~90%) [16], providing some confidence that teachers’ self-reported data were reliable. Teachers were also required to adjust their teaching modalities between in-person and virtual instruction. Anecdotally, many teachers reported greater difficulty in interacting with students virtually. Students were less engaged and attendance decreased. Further, virtual instruction made it difficult for school coordinators to monitor FOYC+CImPACT sessions and for mentors to provide SAM. More training on using the virtual platform to engage students and monitor sessions may have benefitted teachers. König et al. noted the importance of teachers’ digital competency for online instruction [21]. Where schools relied on virtual platforms in The Bahamas, teachers were forced to develop some level of proficiency with those platforms, but more training could contribute to future success. Virtual platforms could provide more opportunities for parents to join the CImPACT session and for teachers to attend training webinars by reducing travel and time costs of parents and teachers. Although disparities in Internet access need to be addressed in The Bahamas, virtual platforms offer many potential benefits for education.

## Conclusion

It is common to direct humanitarian efforts toward disaster relief in times of crisis. However, interruptions to education do not diminish the importance of EBIs such as FOYC+CImPACT. In fact, some data indicate that safe-sex programing may be more important after a natural disaster [16]. Nevertheless, HIV prevention remains an important goal in The Bahamas and worldwide. Our findings highlight teacher supports that can promote implementation fidelity of school-based EBI when educational programing resumes virtually and/or in-person. Teacher training that increases self-efficacy and school coordinators that monitor and provide feedback on their implementation can enhance teachers’ implementation fidelity. Planning for such measures can strengthen programs’ sustainability in the face of the ongoing COVID-19 pandemic and future public health emergencies.

## Data Availability

Data and materials are available for upon request.

## Abbreviations

BMF: Biweekly monitoring and feedback
CImPACT: Caribbean Informed Parents and Children Together
EBIs: Evidence-based intervention
EPIS: Exploration, Preparation, Implementation, and Sustainment
FOYC: Focus on Youth in the Caribbean
HFLE: Health and Family Life Education
MOE: Bahamian Ministry of Education
MOH: Bahamian Ministry of Health
SAM: Site-based assistance and mentorship
VIF: Variance inflation factors
